# SNHG1 promotes malignant biological behaviors of glioma cells via microRNA-154-5p/miR-376b-3p- FOXP2- KDM5B participating positive feedback loop

**DOI:** 10.1186/s13046-019-1063-9

**Published:** 2019-02-06

**Authors:** Han Li, Yixue Xue, Jun Ma, Lianqi Shao, Di Wang, Jian Zheng, Xiaobai Liu, Chunqing Yang, Qianru He, Xuelei Ruan, Zhen Li, Yunhui Liu

**Affiliations:** 1 Department of Neurosurgery, Shengjing Hospital of China Medical University, Shenyang, Liaoning 110004 People’s Republic of China; 2 Liaoning Clinical Medical Research Center in Nervous System Disease, Shenyang, Liaoning 110004 People’s Republic of China; 3 Key Laboratory of Neuro-oncology in Liaoning Province, Shenyang, Liaoning 110004 People’s Republic of China; 4 Department of Neurobiology, College of Basic Medicine, China Medical University, Shenyang, Liaoning 110122 People’s Republic of China; 5 Key Laboratory of Cell Biology, Ministry of Public Health of China, China Medical University, Shenyang, Liaoning 110122 People’s Republic of China; 6 Key Laboratory of Medical Cell Biology, Ministry of Education of China, China Medical University, Shenyang, Liaoning 110122 People’s Republic of China

**Keywords:** Long non-coding RNA, microRNA, Transcription factor, Glioma, Oncogenes

## Abstract

**Background:**

Long non-coding RNAs has been reported in tumorigenesis and play important roles in regulating malignant behavior of cancers, including glioma.

**Methods:**

According to the TCGA database, we identified SNHG1, miRNA-154-5p and miR-376b-3p whose expression were significantly changed in the glioma samples. Furthermore, we investigated SNHG1, miRNA-154-5p and miR-376b-3p expression in clinical samples and glioma cell lines using qRT-PCR analysis and the correlation between them using RNA immunoprecipitation and dual-luciferase reporter. The underlying mechanisms of SNHG1 in glioma were also investigated using immunohistochemistry staining, Western blotting, chromatin immunoprecipitation, and RNA pulldown. Cell Counting Kit-8, transwell assays, and flow cytometry were used to investigate malignant biological behaviors.

**Results:**

We have elucidated the potential molecular mechanism of long non-coding RNA SNHG1 regulating the malignant behavior of glioma cells by binding to microRNA-154-5p or miR-376b-3p. Moreover, our deep-going results showed that FOXP2 existed as a direct downstream target of both microRNA-154-5p and miR-376b-3p; FOXP2 increased promoter activities and enhanced the expression of the oncogenic gene KDM5B; and KDM5B also acts as a RNA-binding protein to maintain the stability of SNHG1.

**Conclusion:**

Collectively, this study demonstrates that the SNHG1- microRNA-154-5p/miR-376b-3p- FOXP2- KDM5B feedback loop plays a pivotal role in regulating the malignant behavior of glioma cells.

**Graphical abstract:**

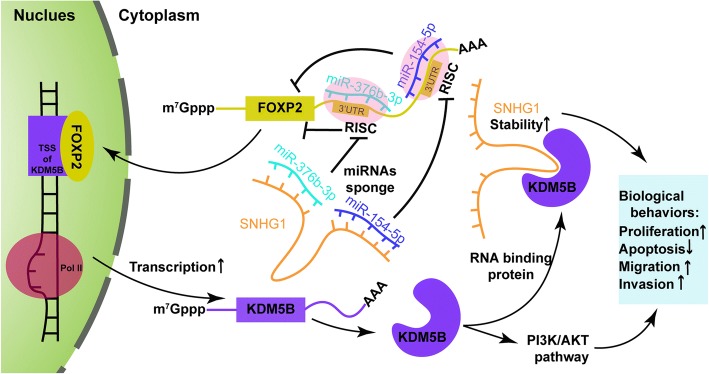

**Electronic supplementary material:**

The online version of this article (10.1186/s13046-019-1063-9) contains supplementary material, which is available to authorized users.

## Background

Glioma is the most common primary brain tumor in human adults. The prognosis of glioma patients is still very poor to date, despite that surgery, radiotherapy, and chemotherapy in glioma treatment are improving [1]. Current studies show that due to the fact that coding genome accounts for less than 2% of all sequences, which is not merely sufficient to elucidate the molecular mechanism of glioma formation and malignant disorders. In addition to coding genome, the dysregulation of non-coding RNA -- which accounts for the vast majority of genomic sequences -- is proposed to affect the development of tumors [2, 3].

Long non-coding RNAs and miRNAs are all classical non-coding RNAs. Numerous studies have found that lncRNAs and miRNAs play an important roles in regulating the development of glioma [4–6]. In the studies of several malignant tumor tissues, it has been found that small nucleolar RNA host gene 1(SNHG1), is abnormally high expressed which is closely related to malignant progression and poor prognosis of tumor [7–10]. In a recent glioma study, it has also been discovered that the expression of SNHG1 can reduce the proliferation and invasion of glioma cells, resulting in more cell apoptosis. This increase in the SNHG1 expression is associated with poor prognosis, however, the molecular mechanisms underlying the biological effects of SNHG1 have not been well understood [11]. SNHG1 can promote tumor growth by regulating the transcription of proximal and distal genes [12]. We predict that many miRNAs are associated with SNHG1 by using bioinformatics methods. Among those SNHG1-associated miRNAs, miR-154-5p and miR-376b-3p were identified. Many studies have shown that miR-154-5p can act as a tumor suppressor via inhibiting the proliferation and metastasis of glioblastoma cells through PIWIL1 binding, which can be used to predict the prognosis of glioma patients. Similar tumor suppression effects by miR-154-5p were also found in other tumor cell lines [13–16]. It also has been shown that miR-376b is involved in development of malignant tumors, such as breast cancer and other tissue cancers [17, 18]. Similar to SNHG1, the miRNA-376 family -- including miRNA-376a, miRNA-376b, and miRNA-376c -- can be individually used as a biomarker for the diagnosis and prognosis evaluation in glioma [19]. Moreover, the miRNA-376 family clusters can produce specific A to I editing in brain tissue, changing the originally targeted silencing genes, and thus, leading to a change in the invasive ability of malignant glioma cells [20, 21].

Fork-head box protein P2 (FOXP2) gene belongs to the fork-head box transcription factor family. The FOXP2 gene is expressed in many tissues and especially plays an important role in the process of brain development and maturation [22]. FOXP2 is involved in the occurrence and development of many other tumor tissues, and whether FOXP2 is a tumor suppressor or tumor-promoting gene remains controversial [23–26]. The relationship between FOXP2 and glioma has not been reported yet.

Lysine-specific demethylase 5B (KDM5B) is a histone lysine specific demethylase, also known as Jarid1B or PLU1. KDM5B can demethylate H3K4me3 and H3K4me2 at lysine 4 on the histone H3 protein, thereby KDM5B regulates individual epigenetic change [27, 28]. Many studies confirm that KDM5B, as a tumor promoting factor, is involved in the tumorigenesis of multiple tissues [29–31]. Overexpression of KDM5B can enhance the tumorigenicity and drug resistance of neuroblastoma in the nervous system diseases [32]. KDM5B can exert biological effects through downstream signaling pathways such as E2F/RB and PI3K/AKT/mTOR. For instance, inhibition of KDM5B expression in bladder and lung tumor cells can inhibit tumor suppressor by down regulating E2F/RB pathway related proteins [33, 34].

In this study, we intially identified the endogenous expression of SNHG1, miR-154-5p, miR-376b-3p, FOXP2, and KDM5B in gliomas. We then studied their important roles in the biological behaviors of glioma cells. We further investigated whether SNHG1 can control the expression of FOXP2 and KDM5B by regulating the expression of miR-154-5p and miR-376b-3p to understand their potential mechanism in regulating biological behaviors of glioma cells and lay down a new fundation for targeted therapy aiming to cure human glioma.

## Methods

### Patients’ tissue samples

Glioma tissues and normal brain tissues (NBTs) were collected from patients undergoing surgery at the Department of Neurosurgery, Shengjing Hospital of China Medical of University from January 2016 to December 2016, after they provided written informed consent. Samples were processed following the standard operation and storage procedures with appropriate ethical approval by the Research Ethics Committee of Shengjing Hospital. Glioma samples were divided into two groups: low-grade glioma group (LGG, WHO I–II, *n* = 10) and high-grade glioma group (HGG, WHO III–IV, *n* = 13).

### Cell culture

Human GBM cell lines (U87 and U251) cells were obtained from Shanghai Institutes for Biological Sciences Cell Resource Center, grown in Dulbecco’s Modified Eagle Medium (DMEM) of high glucose with 10% fetal bovine serum (FBS, Gibco, Carlsbad, CA, USA). Human normal astrocytes and human embryonic kidney (HEK) 293 T were cultured in RPMI 1640 Medium (Gibco, Carlsbad, CA, USA). All cells were maintained in a humidified incubator at 37 °C with 5% CO2.

### Online data extraction and analysis from cancer database and bioinformatics tools

The low-grade glioma (LGG) and glioblastoma multiforme (GBM) IlluminaHiSeq RNAseq datasets were extracted from The Cancer Genome Atlas (TCGA, https://cancergenome.nih.gov) and analysed through R project for statistical computing. Starbase (http://starbase.sysu.edu.cn/index.php) and Targetscan (http://www.targetscan.org) are used to predict binding sites of miR-154-5p and miR-376b-3p. To predict the functional association between KDM5B and SNHG1, RPISeq (http://pridb.gdcb.iastate.edu/RPISeq/) is used to assess the propensity for KDM5B- SNHG1 interaction. Furthermore, catRAPID RNA-Protein interaction validation tool (http://s.tartaglialab.com/page/catrapid_group) is also employed to estimate their interaction strength. The online websites mentioned above were freely accessible.

### Reverse transcription and quantitative real-time polymerase chain reactions (qRT-PCR)

One-Step SYBR PrimeScript RT-PCR Kit (Takara Bio, Inc., Japan) was used for qRT-PCR detection of SNHG1 and mRNA of FOXP2, KDM5B and GAPDH. 10 nanograms of the total RNA containing microRNA was reverse transcribed to cDNA using a TaqMan MicroRNA Reverse Transcription Kit (Applied Biosystems, Foster City, CA, USA). qRT-PCR was conducted using TaqMan Universal Master Mix II with TaqMan microRNA assays of miR-154-5p, miR-376b-3p and U6. GAPDH and U6 were used as the internal control. The expression of each gene was quantified by measuring cycle threshold (Ct) values and normalized using the 2^-∆∆Ct^ method relative to GAPDH or U6.

### Cell transfections

Short-hairpin RNA directed against human SNHG1, FOXP2 and KDM5B was constructed in pGPU6/GFP/Neo vector (sh-SNHG1, sh-FOXP2 and sh-KDM5B), their respective non-targeting sequence (negative control, NC) were also synthesized (GenePharma, Shanghai, China). FOXP2 full length with and without 3’UTR plasmids and their non-targeting sequence (negative control, NC) were synthesized (GenScript, Piscataway, NJ, USA). U87 and U251 cells was transfected at about 70–80% confluency in 24-wells plates using Lipofectamine 3000 reagents (Invitrogen, CA, USA) according to the manufacturer’s instructions. Stable transfected cells were obtained by continuous application of Geneticin (G418, Invitrogen, CA, USA) and evaluated for their over-expression and silence efficiencies by qRT-PCR.

The oligonucleotide sequences of human miR-154-5p and miR-376b-3p mimics, as well as miR-154-5p and miR-376b-3p inhibitors were synthesized (GenePharma, Shanghai, China). A scrambled sequence was used as the negative control. Furthermore, miR-376b-3p + 44 A to I editing mimics were synthesized (GenePharma, Shanghai, China). Cells were transfected using Lipofectamine 3000 reagent (Invitrogen, CA, USA). The transfected efficacy was evaluated by qRT-PCR, and the high transfection efficacy of these could sustain 7 days from 48 h post-transfection.

### Cell proliferation assay

Cell proliferation was performed using Cell Counting Kit-8 (CCK-8, Beyotime Institute of Biotechnology, Jiangsu, China) assay. The cells were seeded in 96-well plates at a density of 2000 cells per well and were treated under different transfection conditions. 48 h later, 10 μl CCK-8 was added to each well. Then, the plates were further cultured in the incubator for another 2 h at 37 C. The absorbance was recorded at 450 nm on a SpectraMax M5 microplate reader (Molecular Devices, USA).

### Flow cytometry analysis

Apoptosis was detected by staining with Annexin V-Fluorescein Isothiocyanate (FITC)/ Propidium Iodide (PI) (BD Biosciences) following the instructions of the manufacturer, then cells were analyzed by flow cytometry (FACScan, BD Biosciences), and the percentage of the cells in different phases was counted.

### Transwell migration and invasion assay

To evaluate the migratory and invasive potential of cells, Transwell chambers with 8-μm pore (Corning, Cambridge, USA) were used. It was specific for the invasion assay that the Transwell chamber was precoated with Matrigel (BD Biosciences, FranklinLakes, NJ, USA) and incubated for 2 h at 37 °C. Cells were resuspended in 200 μL serum-free medium at 2 × 10^5^ cells per mL and injected into the upper compartment. 600 μL of 10% FBS medium was added to the lower compartment. After incubation for 24 h, the cells on the top of membranes were removed, and the cells that penetrated the membrane were fixed and stained with 20% Giemsa. Cells were counted under a microscope in five randomly chosen fields and an average number was calculated.

### Dual-luciferase reporter assay

The fragment of SNHG1 containing the putative miR-154-5p or miR-376b-3p binding sites was subcloned into a pmirGLO Dual-Luciferase miRNA Target Expression Vector (Promega, Madison, WI, USA) to construct the reporter vector SNHG1-wild-type (SNHG1-Wt) (GenePharma, Shanghai, China). Similarly, the corresponding mutants of putative miR-154-5p or miR-376b-3p binding sites were formed to construct the reporter vector SNHG1-mutated-types (SNHG1-Mut) (GenePharma, Shanghai, China). The 3′-UTR fragment of FOXP2 gene and its mutant form of the putative miR-154-5p or miR-376b-3p binding site were subcloned into a pmirGLO vector as mentioned above to form the reporter vector FOXP2–3′UTR-wild-type (FOXP2–3′UTR-Wt) and FOXP2–3′UTR-mutated-type (FOXP2–3′UTR-Mut) (GenePharma, Shanghai, China), respectively. The pmirGLO vector (wild type fragments or mutated type fragments) and indicated miRNAs were transfected into HEK 293 T cells using Lipofectamine 3000. Relative luciferase activities were measured 48 h after transfection and firefly luciferase activity was normalized by renilla luciferase activity.

The responsive FOXP2-binding sites in the KDM5B promotor were determined by dual-luciferase reporter system. Different promoter fragments and human full-length FOXP2 were subcloned into pGL3-basic vector (Promega, Madison, WI, USA) and pEX3 vector (GenePharma, Shanghai, China), respectively. The relative luciferase activity was expressed as the ratio of firefly luciferase activity to renilla luciferase activity.

### Western blot analysis

Cells were lysed in RIPA buffer on ice and total proteins were extracted and separated by SDS-PAGE gels, then electrophoretically transferred to polyvinylidene difluoride membranes. Western blot analysis was performed according to standard procedures. The primary antibodies included anti-FOXP2 (1:1000, ab58599, Abcam), anti-KDM5B (1:2000, ab181089, Abcam) and anti-PI3K, anti-p-PI3K, anti-AKT, anti-p-AKT (1:1000, #4257, #4228, #9272, #9271, Cell Signaling Technology), anti-GAPDH (1:10000, 60,004–1-Ig, Proteintech Group). GAPDH on the same membrane was used as a loading control. Immunoblots were visualized by ECL chemiluminescent detection system. The blots were scanned and the integrated density value (IDV) was measured on FluorChem 2.0 software.

### Immunohistochemistry staining (IHC) assay

For IHC assay, 23 glioma samples (low grade *n* = 10, high grade *n* = 13) were paraffin-embedded and 3 normal brain tissue samples were formalin-fixed. All the samples were cut into 3-μm-thick sections and were stained with hematoxylin and eosin (H&E) to confirm the pathological grade. Immunohistochemistry was performed with standard methodology as previously described. The primary antibody anti-FOXP2 was diluted to 1:100 (ab58599, Abcam). The scoring method is as follows: the percentage of stained cells (P, where 0: < 10%; 1: 10–49%; 2: 50–89%; and 3: > 90%) and staining intensity (I, where 0: negative; 1: weakly positive; 2: moderately-positive; and 3: strongly positive). Based on the value of ΣPI, the stained sections were defined as having low expression [0 (−) to 1 (+)] or high expression [2 (++) to 3 (+++)]. All the assessments were done in a blinded manner and determined independently by two senior pathologists.

### Chromatin immunoprecipitation (CHIP) assay

ChIP assay was performed using Simple ChIP Enzymatic Chromatin IP Kit (Cell signaling Technology, Danvers, Massachusetts, USA) according to the manufacturer’s protocol. In brief, cells were cross-linked with 1% formaldehyde and collected in lysis buffer. 2% lysates were used as an input control and the remaining lysates were immunoprecipitated with normal rabbit IgG and FOXP2 antibody (ab58599, Abcam). DNA was extracted for PCR amplification of the following DNA fragments: putative binding site 1 using the forward primer 5’-GCGAGAGGGAATCGTAGGAC -3′ and reverse primer 5’-GTGTCATGTGCTCTCTCGGC -3′, yielding a 197 bp product; putative binding site 2 using the forward primer 5’-TTTCTGAGGCTGCTGGACAC -3′ and reverse primer 5′ -ATTTTGCAGAGCAGGCAACG -3′, yielding a 134 bp product; control using the forward primer 5′ -GAAACCCAGAAATGAGCGCC -3′ and reverse primer 5′ -AGCTGGTCCCTTATGGTGGA -3′, yielding a 113 bp product.

### RNA immunoprecipitation (RIP) assay

RIP assay was performed using the EZ-Magna RIP Kit (Millipore, Billerica, MA, USA) following the manufacturer’s protocol. In brief, cells were lysed using RIP buffer and then incubated with RIP buffer containing magnetic beads conjugated with human antibodies (anti-Ago2 or anti-KDM5B) or negative control normal mouse IgG. Anti-SNRNP70 was used as positive control for the RIP procedure. The isolated and purified RNA was further used for qRT-PCR analysis of SNHG1, miR-154-5p and miR-376b-3p.

### RNA pull-down assay

Biotin-labelled SNHG1 and antisense SNHG1 transcripts were synthesized (GenePharma, Shanghai, China) and transfected into 293 T cells. After 48 h, whole cell lysates were harvested. RNA-protein complexes were further isolated by Dynabeads M-280 Streptavidin (Invitrogen, Carlsbad, CA, USA). Biotinylated SNHG1 and antisense SNHG1 were incubated with beads for 10 min, and treated with washing buffer. The recruited proteins were detected by Western blot using KDM5B antibody as described above.

### Half-life assay

Cells were dispensed into each well of a 6-well plate. After 48 h, each well was added with 5 μg/ ml Actinomycin D. At each time point, total RNA of a well was collected by direct addition of Trizol and RNA levels were quantitated by qRT-PCR as mentioned above. Actinomycin treatments were repeated at least twice for each cell line and time point, and qRT-PCR was performed in triplicate.

### In vivo xenograft model in nude mice

The stable expressing cells were applied in the in vivo study. Lentivirus encoding miR-154-5p and miR-376b-3p were generated using pLenti6.3/V5eDEST Gateway Vector Kit (Life Technologies Corporation, Carlsbad, CA, USA). The miRNAs involved and short-hairpin RNA targeting human SNHG1 were ligated into the pLenti6.3/V5eDEST vector and LV3-CMV-GFP- Puro vector (GenePharma, Shanghai, China), respectively. And then pLenti6.3/V5eDEST-miR-154-5p, pLenti6.3/V5eDEST-miR-376b-3p and LV3- CMV-GFPPuro-sh-SNHG1 vectors were generated. The ViraPower Packaging Mix was used to generate Lentivirus in 293FT cells. After infection, the stable expressing cells of miR-154-5p (pre-miR-154-5p), miR-376b-3p (pre-miR-376b-3p), sh-SNHG1 were picked. The lentiviruses of miR-154-5p and miR-376b-3p were both transduced in sh-SNHG1 stably transfected cells to generate sh-SNHG1 + pre-miR-154-5p + pre-miR-376b-3p cells.

The nude mice were divided into five groups: control group, sh-SNHG1 group, pre-miR-154-5p group, pre-miR-376b-3p group and sh-SNHG1+ pre-miR-154-5p + pre-miR-376b-3p group. For subcutaneous implantation, 3 × 10^5^ cells were subcutaneously injected in the right flank of the mice. Tumor volume was measured every 5 days when the tumors were apparently seen and calculated by the formula: volume (mm^3^) = length × width^2^/2. 35 days after implantation, mice were sacrificed and tumors were isolated. For survival analysis in orthotropic transplantation, 3 × 10^5^ cells were stereotactically transplanted into the right striatum of the mice. The number of survived nude mice was recorded every day and survival analysis was conducted applying Kaplan-Meier survival curve.

### Statistical analysis

SPSS 18.0 statistical software was used for statistical analysis. All data are presented as the mean ± standard deviation (SD) from at least three independent replicates. Statistical analysis of data was performed using the Student’s t-test. Differences were considered to be statistically significant when *P* < 0.05.

## Results

### Knockdown of SNHG1 inhibited malignant behaviors of glioma cells

**Fig. 1:**
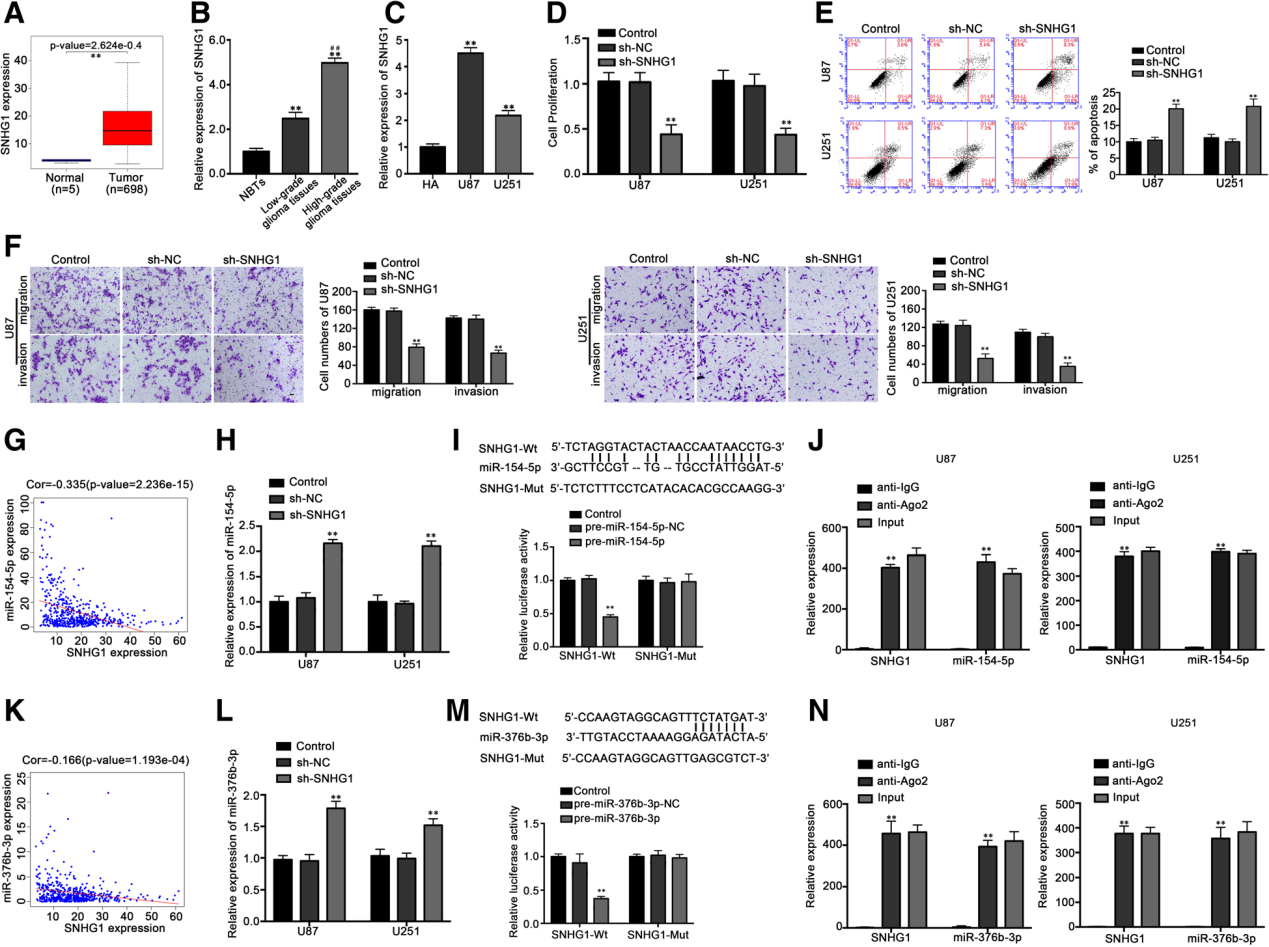
SNHG1 functioned as an oncogene in both glioma tissues and cells and interacted with miR-154-5p and miR-376b-3p. **a** Expression levels of SNHG1 in clinical normal brain (*n* = 5) and glioma samples (LGG and GBM, *n* = 698) were presented as a box- whisker plot. Data were obtained from TCGA data sets. **P < 0.01vs. normal group. **b** Expression of SNHG1 in glioma tissues of different grades and normal brain tissues (NBTs) which were all obtained from clinical cases. Data were presented as the mean ± SD (*n* = 10, each group). **P < 0.01 vs. NBTs group. ^*##*^*P* < 0.01 vs. low grade group. **c** Expression of SNHG1 in HA, U87 and U251 cells. **P < 0.01 vs. HA group. **d** CCK-8 assay was performed to explore the proliferation effect of SNHG1 on U87 and U251 cells. **P < 0.01 vs. sh-NC group. **e** % apoptosis as determined with Annexin V-FITC antibody and PI staining in flow cytometry. **P < 0.01 vs. sh-NC group. **f** Effects of SNHG1 knockdown on cell migration and invasion of U87 and U251 cells. **P < 0.01 vs. sh-NC group. Scale bars represented 20 μm. Relationship of expression levels between SNHG1 and miR-154-5p (**g**) or miR-376b-3p (**k**) in glioma samples from the TCGA database. The x axis indicated expression levels of SNHG1. The y axis indicated expression levels of indicated miRNAs. *P*-values were obtained by Pearson’s correlation coefficient analysis. The red line in the graph indicated linear correlation (*Cor* = − 0.335 and − 0.166, respectively). qRT-PCR analysis revealed the negative correlation between SNHG1 and miR-154-5p (**h**) or miR-376b-3p (**l**) expression in U87 and U251 cells. **P < 0.01 vs. sh-NC group. Schematic representation of the putative binding site between SNHG1 (SNHG1-Wt) and miR-154-5p (**i**) or miR-376b-3p (**m**), and the contrivable mutant sequence (SNHG1-Mut) indicated for the dual-luciferase reporter assay. Renilla/firefly luciferase ratios were calculated and further normalized. **P < 0.01 vs. pre-NC group. RIP assay with anti-IgG, anti-Ago2 or 10% input from cell extracts. Relative expression of SNHG1 and miR-154-5p (**j**) or miR-376b-3p (**n**) in Ago2 relative to normal IgG immunoprecipitates were determined by qRT-PCR. **P < 0.01 vs. anti-IgG group. Except for A, B, G and K, data in others were presented as the mean ± SD (*n* = 5, each group)

According to the TCGA database, the expression of SNHG1 increased significantly in glioma samples in comparison with the normal brain tissue (*P* < 0.01) (Fig. 1a). The qRT-PCR results showed that the expression of SNHG1 in glioma tissues was higher than that in normal brain tissues, and the expression level was positively correlated with the pathological grade of glioma (*P* < 0.01) (Fig. 1b). The expression of SNHG1 in malignant glioma cell lines U87 and U251 is higher than that in HA cells (*P* < 0.01) (Fig. 1c), suggesting that SNHG1 may play a role in promoting the biological behavior of malignant tumor cells in glioma cells.

In order to clarify the biological role of SNHG1 in glioma cells, SNHG1 was stably silenced in malignant glioma cells U87 and U251, and the proliferation ability of the cells was detected by CCK-8 assay after confirming the transfection efficiency by qRT-PCR. Compared with the control group, the proliferation ability of U87 and U251 in sh-SNHG1 group was significantly decreased (*P* < 0.01), as shown in Fig. 1d. At the same time, the apoptosis rate was detected by flow cytometry. The results showed that the apoptosis rate of U87 and U251 cells increased significantly in sh-SNHG1 group (*P* < 0.01), as shown by 1E. The migration and invasion ability of U87 and U251 cells were detected by Transwell assay. The results showed that the migration and invasion ability of sh-SNHG1 cells decreased significantly (*P* < 0.01) (Fig. 1f).

### SNHG1 bound to and attenuated the expression of miR-154-5p and miR-376b-3p

The TCGA database, via the Pearson’s correlation analysis showed that the expression of miR-154-5p and miR-376b-3p were significantly inverse in correlation with SNHG1 (*P* < 0.01) (Fig. 1g, k). The qRT-PCR detection showed that the expression of miR-154-5p and miR-376b-3p in sh-SNHG1 group was significantly higher than that in sh-NC SNHG1 group (*P* < 0.01) (Fig. 1h, l), suggesting that SNHG1 may have potential binding sites and interaction with miR-154-5p and miR-376b-3p, respectively. The detection results of dual-luciferase report show that when pre-miR-154-5p was cotransfected with wild-type SNHG1 plasmid, its luciferase activity was significantly decreased (*P* < 0.01), but cotransfected with mutant SNHG1 plasmids, luciferase activity had no significant change (Fig. 1i); at the same time, when pre-miR-376b-3p was cotransfected with wild-type SNHG1 plasmid, its luciferase activity was significantly decreased (*P* < 0.01), but when cotransfected with mutant SNHG1 plasmids, luciferase activity had no significant change (Fig. 1m). Further RIP test showed that AGO2 antibody enriched SNHG1, miR-154-5p and miR-376b-3p relative to IgG antibody in U87 and U251 cells, and confirmed that they existed in the RISC complex (Fig. 1j, n). The binding sites between SNHG1 and miR-154-5p, as well as between SNHG1 and miR-376b-3p were verified, and the expression of miR-154-5p and miR-376b-3p was down regulated by binding. At the same time, according to references [21], pre-miR-376b-3p + 44 A to site I (G) mutant plasmid was constructed. Referring to the dual luciferase reporter gene assay, the results showed that there was binding site between SNHG1 and pre-miR-376b-3p (A to I edited), and the inhibitory effect is similar to that of the non A to I edited miR-376b-3p (Additional file 1: Figure S1A).

### MiR-154-5p and miR-376b-3p acted as tumor suppressors in glioma cell lines

**Fig. 2:**
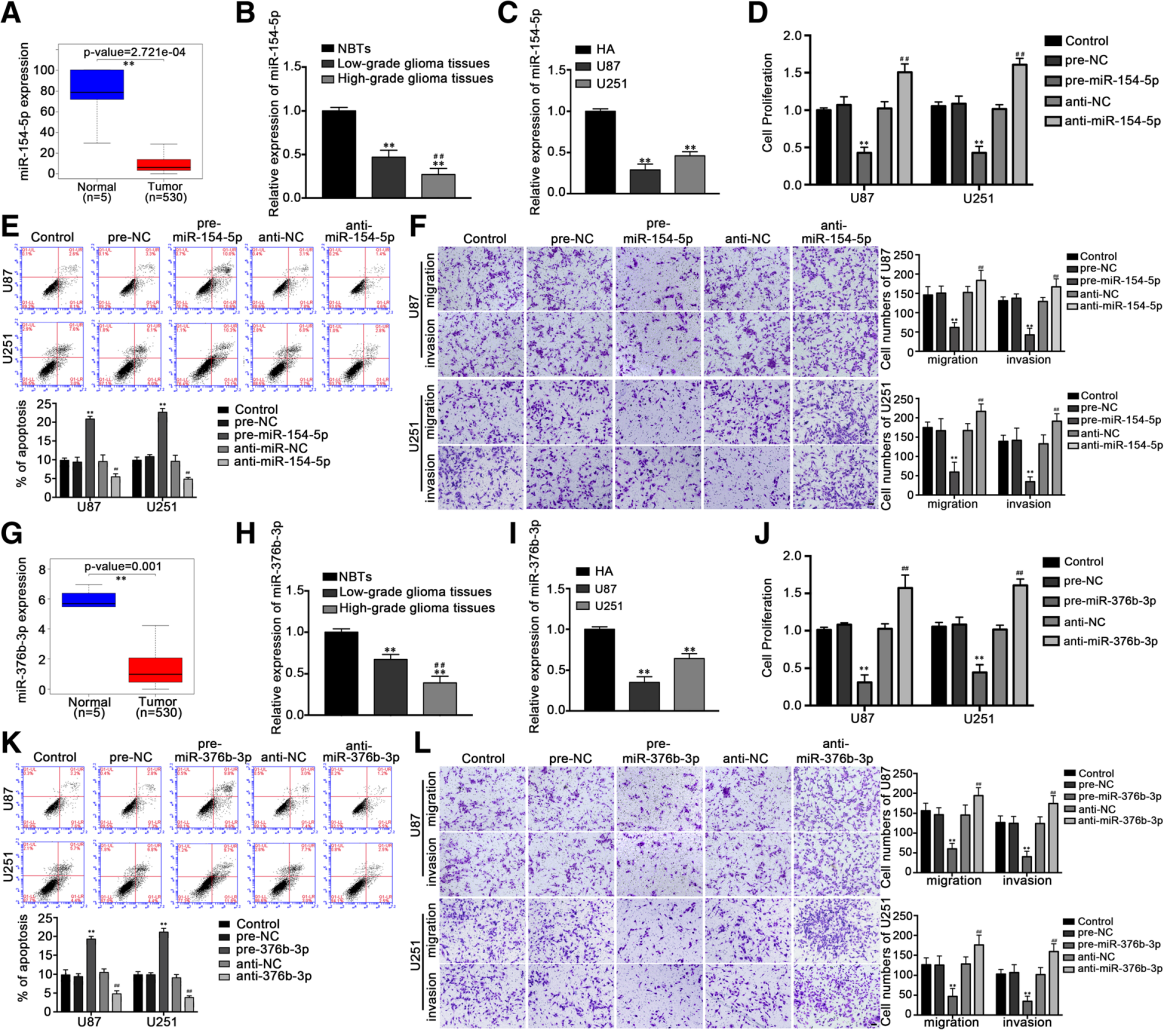
MiR-154-5p and miR-376b-3p manifested an anti-oncogene in both glioma tissues and cells, decreased SNHG1 inhibited the malignant behavior of glioma cells by targeting miR-154-5p and miR-376b-3p. Expression levels of miR-154-5p (**a**) and miR-376b-3p (**g**) in clinical normal brain (*n* = 5) and glioma samples (LGG and GBM, *n* = 530) were presented as a box- whisker plot. Data were obtained from TCGA data sets. **P < 0.01 vs. normal group. Expression of miR-154-5p (**b**) and miR-376b-3p (**h**) in glioma tissues of different grades and normal brain tissues (NBTs) which were all obtained from clinical cases. Expression of miR-154-5p (**c**) and miR-376b-3p (**i**) in HA, U87 and U251 cells. CCK-8 assay was performed to explore the proliferation effect of miR-154-5p (**d**) and miR-376b-3p (**j**) on glioma cells. Flow cytometry analysis of U87 and U251 cells apoptosis with altered miR-154-5p (**e**) and miR-376b-3p (**k**) expression. Effects of miR-154-5p (**f**) and miR-376b-3p (**l**) expression changes on migration and invasion of U87 and U251 cells. Scale bars represented 20 μm. Except for A and G, data above were presented as the mean ± SD (*n* = 5, each group). **P < 0.01 vs. pre-NC group, ^*##*^*P* < 0.01 vs. anti-NC group

According to the TCGA database, the expression of miR-154-5p and miR-376b-3p was significantly decreased in glioma samples in comparison with the normal brain tissue (*P* < 0.01) (Fig. 2a, g); the expression of miR-154-5p and miR-376b-3p in glioma tissues was lower than that in normal brain tissues (*P* < 0.01) by qRT-PCR, and the expression level was negatively correlated with histological grade (Fig. 2b, h). The expression of miR-154-5p and miR-376b-3p in U87 and U251 cells was lower than that in HA cells (*P* < 0.01) (Fig. 2c, i). After overexpression and silencing of miR-154-5p, the results showed that compared with the pre-miR-154-5p-NC group, the proliferation ability, migration and invasion ability of pre-miR-154-5p group decreased (*P* < 0.01), and the apoptosis rate increased significantly (*P* < 0.01); Compared with anti-miR-154-5p-NC group cells, the proliferation ability, migration and invasion ability of anti-miR-154-5p group increased (*P* < 0.01), and the apoptosis rate decreased significantly (*P* < 0.01) (Fig. 2d-f). The same method also detected that in comparison with the pre-miR-376b-3p-NC group, the cell proliferation ability, migration and invasion ability of the pre-miR-376b-3p group decreased (*P* < 0.01), cell apoptosis rate increased significantly (*P* < 0.01); compared with anti-miR-376b-3p-NC group, cell proliferation, migration and invasion ability of anti-miR-376b-3p group increased (*P* < 0.01), the apoptosis rate was significantly lower (*P* < 0.01) (Fig. 2j-l). The results show that miR-154-5p and miR-376b-3p play a role in inhibiting malignant biological behavior in U87 and U251 cells.

### SNHG1 sequestered the tumor-suppressive effects of miR-154-5p and miR-376b-3p on glioma cell lines

In order to clarify the mechanism of SNHG1 promoting the malignant biological behavior of tumor by regulating miR-154-5p and miR-376b-3p, the overexpression and silence plasmids of miR-154-5p and miR-376b-3p were transfected into SNHG1 knockdown U87 and U251 cells. The results showed that compared with sh-NC+ pre-NC group, the cell proliferation, migration ability and invasion ability of sh-SNHG1 + pre-miR-154-5p group were significantly decreased (*P* < 0.01), and the apoptosis rate was significantly increased (*P* < 0.01); There was no significant change in cell biological behavior in sh-SNHG1 + anti-miR-154-5p group. Similarly, compared with the control group, the cell proliferation, migration ability and invasion ability of sh-SNHG1 + pre-miR-376b-3p group were significantly decreased (*P* < 0.01), the apoptosis rate was significantly increased (*P* < 0.01), while the biological behavior of sh-SHNG1 + anti-miR-376b-3p group was not significantly different. Compared with other control groups, the cell proliferation, migration and invasion ability of sh-SNHG1 + pre-miR-154-5p + pre-miR-376b-3p group were significantly decreased (*P* < 0.01), and the apoptosis rate was significantly increased (*P* < 0.01) (Additional file 2: Figure S2A-C).

### FOXP2 acted as an oncogene in glioma cells lines

**Fig. 3:**
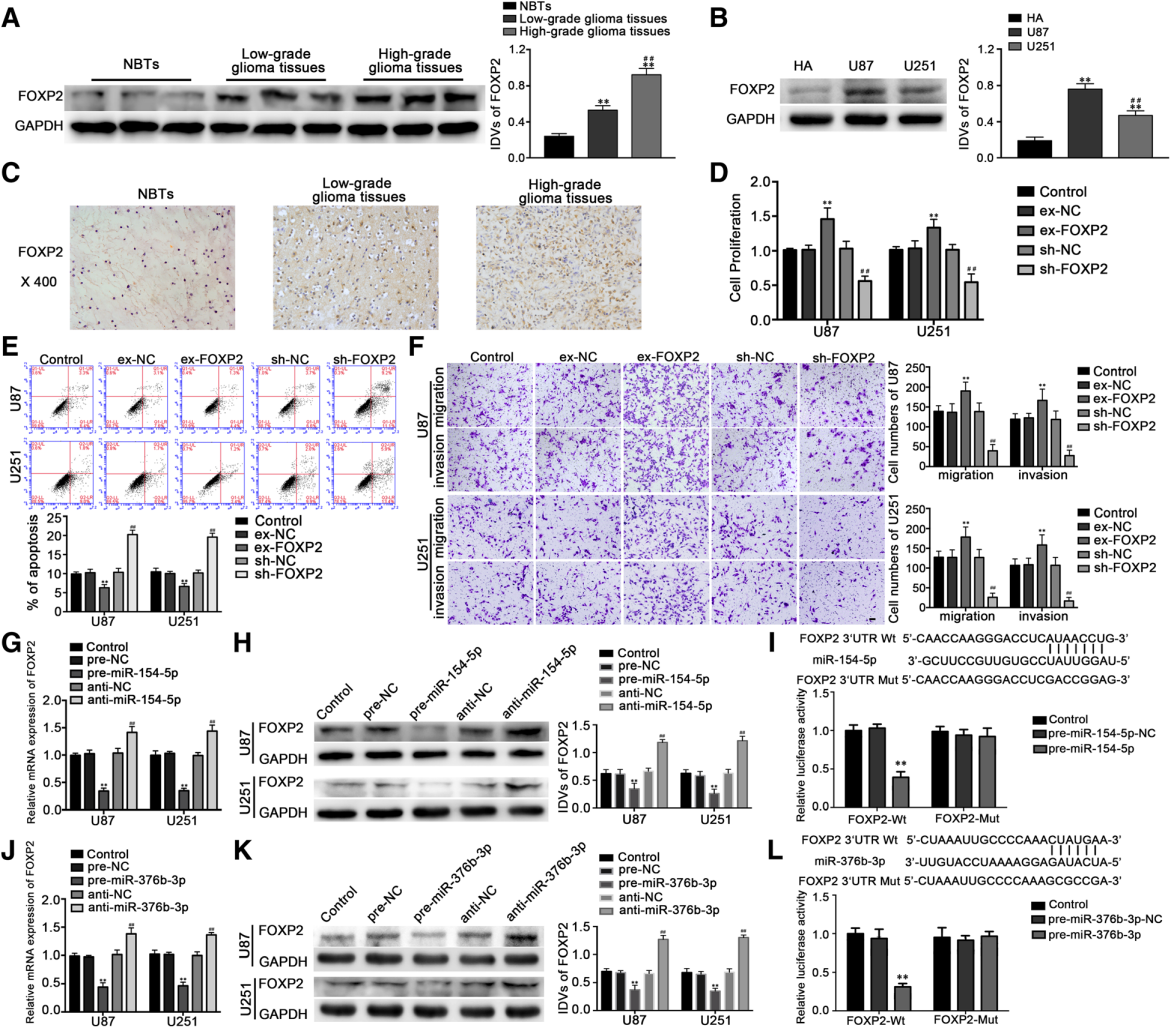
FOXP2 acted as an oncogene in both glioma tissues and cells, miR-154-5p and miR-376b-3p inhibited malignant progression of glioma cells by binding to the FOXP2–3′-UTR. **a** FOXP2 protein expression in glioma tissues of different grades and normal brain tissues (NBTs) with GAPDH as an endogenous control. Data were presented as the mean ± SD (*n* = 10, each group). **P < 0.01 vs. NBTs group. ^*##*^*P* < 0.01 vs. low grade group. **b** Western blot analysis of FOXP2 expression in U87 and U251 cells, with GAPDH as an endogenous control. **P < 0.01 vs. HA group. **c** Representative IHC assay patterns of FOXP2 expression in glioma tissues and normal brain tissues on tissue microarray sections. The photographs were taken at 400× magnification. **d** CCK-8 assay was performed to explore the proliferation effect of FOXP2 on U87 and U251 cells. **e** The apoptosis percentages of U87 and U251 cells were detected after FOXP2 overexpression or inhibition. **f** Effects of FOXP2 expression changes on migration and invasion of U87 and U251 cells. Scale bars represented 20 μm. For B, D, E and F, data were presented as the mean ± SD (*n* = 5, each group). **P < 0.01 vs. ex-NC group, ^*##*^*P* < 0.01 vs. sh-NC group. qRT-PCR and Western blot analysis revealed the negative correlation between miR-154-5p (**g**, **h**) or miR-376b-3p (**j**, **k**) and FOXP2 expression in glioma cells. Data were presented as the mean ± SD (*n* = 5, each group). **P < 0.01 vs. pre-NC group, ^*##*^*P* < 0.01 vs. anti-NC group. For luciferase reporter assay, the predicted miR-154-5p (**i**) or miR-376b-3p (**l**) binding sites in the 3′-UTR region of FOXP2 (FOXP2–3′-UTR-Wt) and the designed mutant sequence (FOXP2–3′UTR-Mut) were indicated. Renilla/firefly luciferase ratios were calculated and further normalized. **P < 0.01 vs. pre-NC group

He [35], one of co-authors, has confirmed that FOXP2 involved in angiogenesis of U87 glioma-exposed endothelial cells and angiogenesis is considered to be one of the mechanisms of tumorigenesis [36]. In this study, the expression of FOXP2 in glioma tissues was higher than that in normal brain tissues; and the expression of high grade pathological tissue was higher than that of low grade; the expression in glioma U87 and U251 cells was significantly higher than that in HA cells, the above differences were statistically significant (*P* < 0.01) (Fig. 3a, b). Immunohistochemistry also showed that the expression of FOXP2 in glioma tissues was higher than that in normal brain tissues, and the expression level increased with the tumor grade. FOXP2 in low grade tumors mainly located in the nucleus, with a small amount in the cytoplasm. In high grade tumors, the distribution of FOXP2 in the cytoplasm increased (Fig. 3c). The FOXP2 immunohistochemical staining results were positively correlated with WHO glioma grade classification (*r* = 0.278, *P* < 0.01) (Table 1).

**Table 1:** Correlation of FOXP2 expression with WHO grade

	Number of patients	FOXP2 staining				Cor value
		–	+	++	+++	
Total	26	4	8	9	5	
Normal tissues	3	2	1			
WHO grade						0.899**
Low grade	10	1	5	4		
High grade	13	1	2	5	5	

^**^P < 0.01 estimated by Spearman’s correlation test

In order to further understand the effect of FOXP2 on the biological behavior of malignant glioma cells, U87 and U251 cells were transfected with FOXP2 overexpression and silence plasmids, and the malignant biological behaviors of U87 and U251 cells were detected. The results show that compared with the ex-FOXP2-NC group, the proliferation, migration and invasion of ex-FOXP2 cells increased, the apoptosis rate decreased; Compared with group sh-FOXP2-NC cells, the cell proliferation, migration, and invasion ability of sh-FOXP2 group decreased, and the apoptosis rate increased, the differences were statistically significant (*P* < 0.01). These results suggest that FOXP2 can promote the malignant biological behavior of glioma cells (Fig. 3d-f).

### MiR-154-5p and miR-376b-3p inhibited FOXP2 expression by targeting its 3’UTR, in turn produced tumor suppressor effects

Bioinformatics software (Starbase, Targetscan) predicted that binding sites existed in miR-154-5p and miR-376b-3p correlating with FOXP2. In order to clarify the mechanism of miR-154-5p and miR-376b-3p regulating the malignant biological behavior of tumor by regulating FOXP2, after overexpression and silencing of miR-154-5p in U87 and U251 cells, by using qRT-PCR and the Western blot methods to detect the expression of FOXP2, the results showed that compared with the pre-miR-154-5p-NC group, the expression of FOXP2 was significantly decreased in pre-miR-154-5p group (*P* < 0.01). Compared with anti-miR-154-5p-NC group, the expression of FOXP2 in anti-miR-154-5p group was significantly increased (*P* < 0.01) (Fig. 3g, h), which proved that miR-154-5p could inhibit the expression of FOXP2 mRNA and protein. The same method proved that the overexpression of miR-376b-3p could inhibit the expression of FOXP2, and silencing miR-376b-3p promoted the expression of FOXP2, indicating that miR-376b-3p could inhibit the expression of FOXP2 mRNA and protein (*P* < 0.01) (Fig. 3j, k). The results of dual luciferase assay showed that the luciferase activity of cotransfected wild-type FOXP2–3 ‘UTR plasmid and pre-miR-154-5p group was significantly decreased, and there was statistical difference (*P* < 0.01); The luciferase activity of pre-miR-154-5p NC group and mutant FOXP2–3 ‘UTR group did not change compared with that of the control group, indicating that there was a binding site between miR-154-5p and FOXP2 mRNA (Fig. 3i). The dual luciferase assay showed that FOXP2 and miR-376b-3p also had binding sites (*P* < 0.05) (Fig. 3l). By applying the dual luciferase experiment of A to I (G) mutant plasmid at pre-miR-376b-3p + 44 locus again, the results showed that there were also binding sites between FOXP2 and pre-miR-376b-3p (A to I edited), and the inhibitory effect is similar to that of the non A to I edited miR-376b-3p (Additional file 1: Figure S1B).

In order to further confirm the mechanism of interaction between miR-154-5p and miR-376b-3p with FOXP2, the miR-154-5p overexpression, miR-376b-3p overexpression, 3 ‘UTR wild-type and 3’ UTR mutant FOXP2 overexpression plasmids were transfected into U87 and U251 cell groups. The results showed that the pre-miR-154-5p + FOXP2–3 ‘UTR-Mut group and the pre-miR-376b-3p + FOXP2–3’ UTR-Mut group produced the same effects like overexpression of FOXP2, which promoted the proliferation, migration, and invasion of glioma cells (*P* < 0.01), and reduced the apoptosis rate of glioma cells (*P* < 0.01). The overexpression of 3 ‘UTR wild-type FOXP2 group reversed the overexpression of miR-154-5p in inhibiting proliferation, migration, and invasion of glioma cells, and reduced the apoptosis rate of glioma cells induced by overexpression of miR-154-5p (*P* < 0.01) (Additional file 2: Figure S2D-F). Similarly, the overexpression of 3 ‘UTR wild-type FOXP2 group reversed the overexpression of miR-376b-3p in inhibiting proliferation, migration, and invasion of glioma cells, and reduced the apoptosis rate of glioma cells induced by overexpression of miR-376b-3p (*P* < 0.01) (Additional file 2: Figure S2D-F). These results confirm that miR-154-5p and miR-376b-3p play a role in inhibiting malignant biological behavior of glioma by binding to FOXP2 mRNA 3’UTR.

### FOXP2 bound to oncogene KDM5B promotors and facilitated its expression

**Fig. 4:**
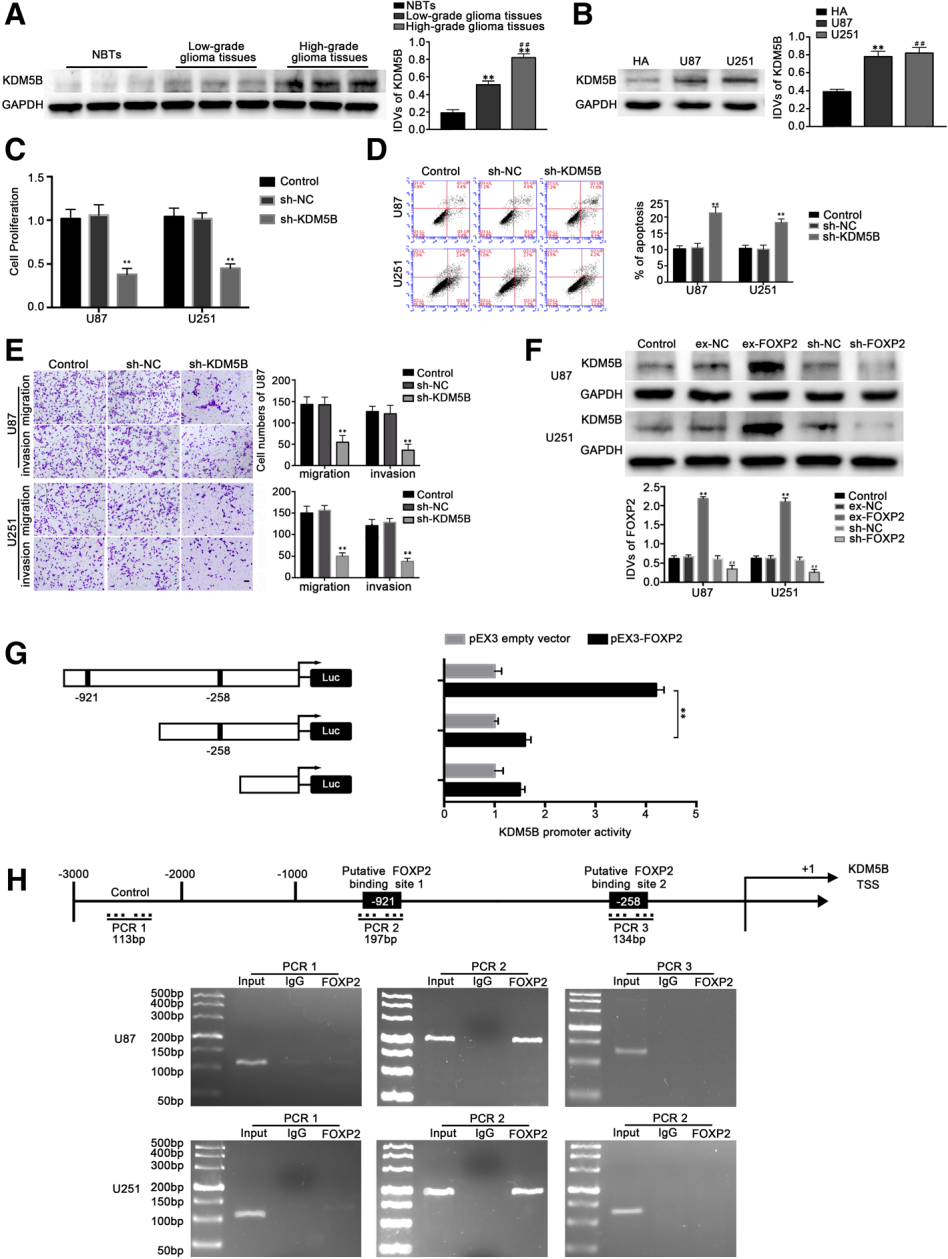
KDM5B functioned as an oncogene in both glioma tissues and cells, FOXP2 mediated malignant progression of glioma cells by targeting transcription start site of KDM5B. **a** KDM5B protein expression in glioma tissues of different grades and normal brain tissues (NBTs) with GAPDH as an endogenous control. Data were presented as the mean ± SD (*n* = 10, each group). **P < 0.01 vs. NBTs group. ^*##*^*P* < 0.01 vs. low grade group. **b** Western blot analysis of KDM5B expression in U87 and U251 cells, with GAPDH as an endogenous control. **P < 0.01 vs. HA group. **c** CCK-8 assay was performed to explore the proliferation effect of FOXP2 on U87 and U251 cells. **d** Incidence of apoptotic cells was studied by flow cytometry. **e** Effects of FOXP2 expression changes on cell migration and invasion of U87 and U251 cells. Scale bars represented 20 μm. For C, D and E, data were presented as the mean ± SD (*n* = 5, each group). **P < 0.01 vs. sh-NC group. **f** Western blot analysis revealed the negative correlation between FOXP2 and KDM5B expression in U87 and U251 cells. Data are presented as the mean ± SD (*n* = 5, each group). **P < 0.01 vs. ex-NC group, ^*##*^*P* < 0.01 vs. sh-NC group. **g** Schematic depiction of the KDM5B reporter constructs and their luciferase activities. The Y-bar showed the position of the deletions on the DNA fragments. X-bar showed the constructed plasmid activity after normalization with the co-transfected reference vector (pRL-TK), and relative to the activity of pEX3 empty vector, which the activity was set to 1. Data represent means ± SD (*n* = 5, each). **h** Schematic representation of the KDM5B promoter region 3000 bp upstream of the transcription start site (TSS) which designated as + 1. ChIP PCR products for putative binding sites and an upstream region not expected to associate with FOXP2 were depicted with bold lines. Immunoprecipitated DNA was amplified by PCR. Images are representative of independent experiments (*n* = 4)

The expression of KDM5B in glioma tissues was higher than that in normal brain tissues, while the expression of high grade pathological tissue was higher than that of low grade, and expression in glioma U87 and U251 cells was significantly higher than that in HA cells. The above differences were statistically significant (*P* < 0.01) (Fig. 4a, b).

In order to further understand the role of KDM5B in glioma, U87 and U251 cells were transfected with KDM5B silencing plasmids, and the malignant biological behaviors of U87 and U251 cells were detected. The results showed that compared with group sh-NC, the cell proliferation, migration, and invasion ability of the sh-KDM5B group were decreased, and the apoptosis rate was increased. The above differences were statistically significant (*P* < 0.01) (Fig. 4c-e). These results suggest that KDM5B can promote the proliferation, migration and invasion of U87 and U251 cells, and inhibit the apoptosis of U87 and U251 cells, which is a tumor promoting gene.

Bioinformatics software (JASPAR) predicted binding sites between FOXP2 and KDM5B promoter region in order to study the mechanism of interaction between FOXP2 and KDM5B. Our study overexpressed and silenced FOXP2 in U87 and U251 cells, then we detected the expression of KDM5B via the Western blot method. The results showed that compared with the ex-FOXP2-NC group, the expression of KDM5B in the ex-FOXP2 group was significantly increased. Compared with the sh-FOXP2-NC group, the expression of KDM5B in the sh-FOXP2 group was significantly decreased (*P* < 0.01) (Fig. 4f), which proved that FOXP2 could promote the expression of KDM5B protein. In order to verify whether FOXP2 is required for KDM5B promoter activity, we identified two FOXP2 binding sites by analyzing these DNA sequences in the upstream 3000 bp region of the KDM5B transcription start site (TSS) and the downstream 100 bp region. Thus, we constructed a wild type PGL3 double luciferase reporter gene vector and one that lacks binding site. The loss of − 921 site significantly reduced the promoter activity of KDM5B after cotransfection with ex-FOXP2 (*P* < 0.01). The results showed that FOXP2 could bind to the − 921 region of KDM5B TSS and stimulate its expression (Fig. 4g). Furthermore, chromatin immunoprecipitation (CHIP) assay was used to verify the binding between FOXP2 and KDM5B promoter region, the upstream 2000 bp region of the predicted FOXP2 binding site was amplified by PCR method, and the negative control was not combined with FOXP2. The results showed that the predicted binding site 1 of FOXP2 had a direct binding effect with KDM5B (Fig. 4h).

### SNHG1 regulates the expression of FOXP2 by inhibiting miR-154-5p and miR-376b-3p, further regulating the expression level of KDM5B

**Fig. 5:**
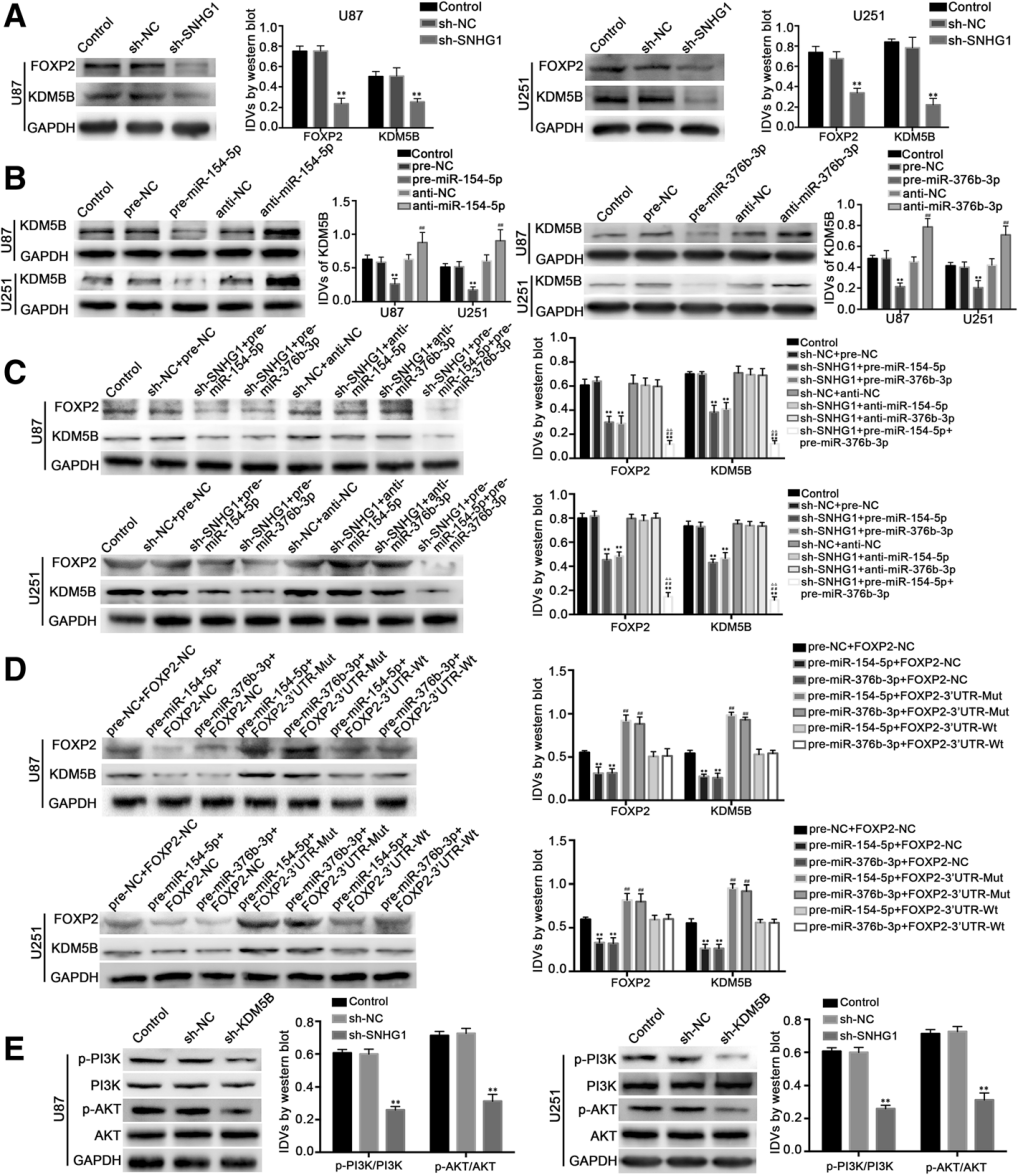
SNHG1 promoted the expression of FOXP2 by inhibiting miR-154-5p and miR-376b-3p, further enhancing the expression level of KDM5B. **a** Western blot analysis on FOXP2 and KDM5B in SNHG1-knockdown glioma cells. **P < 0.01 vs. sh-NC group. **b** Western blot analysis revealed the negative correlation between miR-154-5p or miR-376b-3p and KDM5B expression in U87 and U251 cells. **P < 0.01 vs. pre-NC group, ^*##*^*P* < 0.01 vs. anti-NC group. **c** SNHG1 knockdown and miR-154-5p or miR-376b-3p overexpression decreased the protein expression of FOXP2 and KDM5B in U87 and U251 cells. **P < 0.01 vs. sh-NC + pre-NC group, ^*##*^*P* < 0.01 vs. sh-SNHG1 + pre-miR-154-5p group, ^∆∆^*P* < 0.01 vs. sh-SNHG1 + pre-miR-376b-3p group. **d** FOXP2–3′-UTR-Wt reversed overexpression of miR-154-5p and miR-376b-3p induced attenuation of FOXP2 and KDM5B expression in U87 and U251 cells. **P < 0.01 vs. pre-NC + FOXP2-NC group, ^*##*^*P* < 0.01 vs. pre-miR-154-5p + FOXP2-NC group, ^∆∆^*P* < 0.01 vs. pre-miR-376b-3p + FOXP2-NC group. **e** Western blot analysis of the PI3K/AKT pathway regulated by KDM5B in U87 and U251 cells. **P < 0.01 vs. sh-NC group. Data above were presented as the mean ± SD (*n* = 5, each group). GAPDH was used as an endogenous control

After silencing SNHG1 in U87 and U251, compared with sh-NC group cells, the protein expression of FOXP2 and KDM5B decreased significantly (*P* < 0.01) (Fig. 5a). It is proved that silencing SNHG1 can inhibit the expression of FOXP2 protein and regulate the expression of KDM5B protein. The results suggest that the expression level of FOXP2 is associated with SNHG1, and may be involved in the mechanism of SNHG1 on the biological behavior of malignant glioma cells.

In the overexpression and silencing of miR-154-5p and miR-376b-3p in U87 and U251 cells, through the Western blot method detecting the protein expression of KDM5B, the results showed that compared with pre-NC group, the expression of KDM5B was significantly decreased in pre-miR-154-5p and pre-miR-376b-3p groups, compared with the anti-NC group, the expression of KDM5B in anti-miR-154-5p group and anti-miR-376b-3p group was significantly higher (*P* < 0.01) (Fig. 5b). Further in the SNHG1 knockdown U87 and U251 cells cotransfected with miR-154-5p overexpression and silence plasmid, then via the Western blot method detecting the protein expression of FOXP2 and KDM5B, the results showed that the protein expression of FOXP2 and KDM5B in sh-SNHG1 + pre-miR-154-5p group decreased significantly (*P* < 0.01), while silencing miR-154-5p (sh-SNHG1 + anti-miR-154-5p) could counteract the inhibition of FOXP2 and KDM5B protein expression. The protein expression of FOXP2 and KDM5B decreased significantly when SNHG1 knockdown U87 and U251 cells were cotransfected with both miR-154-5p and miR-376b-3p overexpression plasmids (*P* < 0.01) (Fig. 5c). It is proved that miR-154-5p and miR-376b-3p are involved in the SNHG1 regulating transcription factor FOXP2 and downstream protein KDM5B.

The detection of downstream protein KDM5B was applied to the 7 groups of glioma cells transfected with miR-154-5p overexpression, miR-376b-3p overexpression, 3 ‘UTR wild-type and 3’ UTR mutant FOXP2 overexpression plasmid. The results showed that in the group of over expression of 3 ‘UTR mutant FOXP2, KDM5B protein expression was significantly increased (*P* < 0.01) (Fig. 5d). The overexpression of 3 ‘UTR wild-type FOXP2 group could counteract the inhibitory effect of miR-154-5p or miR-376b-3p on the expression of KDM5B protein (*P* < 0.01) (Fig. 5d). This further proved that miR-154-5p and miR-376b-3p regulate the expression of downstream protein KDM5B by binding to the 3 ‘UTR specific sequence of transcription factor FOXP2.

In addition, the Western blot assay also showed that the levels of p-PI3K/PI3K and p-Akt/Akt changed in stably transfected and silenced KDM5B U87 and U251 cells. Compared with group ex-NC, p-PI3K/PI3K and p-Akt/Akt increased significantly in group ex-KDM5B; compared with group sh-NC cells, p-PI3K/PI3K, and p-Akt/Akt decreased significantly in sh-KDM5B group, the differences were statistically significant (*P* < 0.01) (Fig. 5e), it is concluded that KDM5B can regulate the biological behavior of glioma cells by activating the PI3K/Akt pathway.

### KDM5B bound to SNHG1 and formed a positive feedback loop

**Fig. 6:**
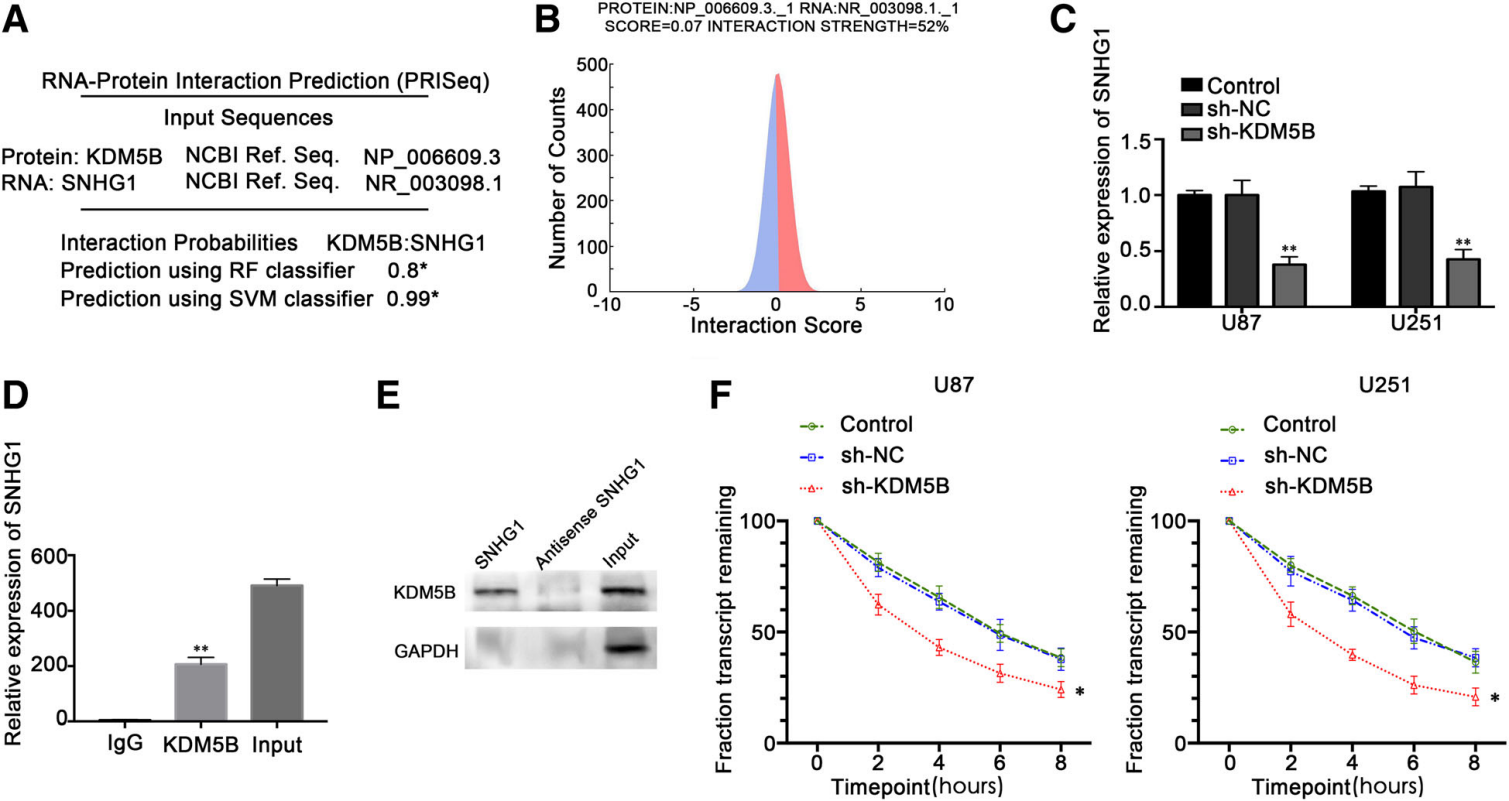
KDM5B bound to SNHG1 and formed a positive feedback loop to promote the biological behavior of glioma cells. **a** Sequence-based prediction of KDM5B-SNHG1 interaction, with random forest, RF classifier score of 0.8 and support vehicle machine, SVM classifier score of 0.99. * Interaction probabilities generated by RPISeq range from 0 to 1. In performance evaluation experiments, predictions with probabilities > 0.5 were considered “positive” indicating that the corresponding protein and RNA are likely to interact. **b** Interaction strength based on interaction propensity rank of binding regions in the positive set using catRAPID strength. **c** qRT-PCR displayed positive correlation between KDM5B and SNHG1. Data were presented as the mean ± SD (*n* = 5, each group). **P < 0.01 vs. sh-NC group. **d** RIP assay with anti-IgG, anti-KDM5B or 10% input from cell extracts. Relative expression of SNHG1 in KDM5B relative to normal IgG immunoprecipitates were determined by qRT-PCR. **P < 0.01 vs. anti-IgG group. **e** RNA-pulldown assay was performed using in vitro synthesized biotinylated SNHG1. Precipitation reactions were conducted using streptavidin beads and then subjected to Western blot. **f** SNHG1 RNA half-life measured by qRT-PCR after Actinomycin D treatment. Data were presented as the mean ± SD (*n* = 3, each group). *P < 0.05 vs. sh-NC group

Bioinformatics software (RPISeq) predicted that there was a strong interaction propensity between KDM5B and SNHG1 (Fig. 6a). Furthermore, catRAPID predicted that KDM5B may have a stable binding to SNHG1 (Fig. 6b). In order to determine whether there was a loop between KDM5B and SNHG1, the expression of SNHG1 was detected by qRT-PCR in KDM5B knockdown U87 and U251 cells. The results showed that compared with sh-NC group, the expression of SNHG1 in sh-KDM5B group was significantly decreased (*P* < 0.01) (Fig. 6c). Thus, proving that silencing KDM5B could also inhibit the expression of SNHG1. Further experiments with RIP confirmed that KDM5B could specifically bind to SNHG1. The results of RIP-PCR showed that the amount of SNHG1 enriched by KDM5B protein was significantly higher than that in IgG group (*P* < 0.05) (Fig. 6d), continuing the pull-down experiments, the Western blot method showed that KDM5B protein could be identified in lncRNA SNHG1 sense strand group, while in lncRNA SNHG1 antisense strand group, KDM5B protein was not identified, and further verified that there was a specific binding between KDM5B and SNHG1 (Fig. 6e). QRT-PCR was used to detect the inhibition of actinomycin D on the half-life of SNHG1 after transcription. The results showed that the half-life of SNHG1 in sh-KDM5B transfected cells was shortened, and the degradation of SNHG1 was accelerated significantly compared with the control group. KDM5B could delay the degradation time of SNHG1 after transcription (Fig. 6f).

### SNHG1 knockdown combined with miR-154-5p and miR-376b-3p overexpression suppressed tumor growth in xenograft nude mice model

**Fig. 7:**
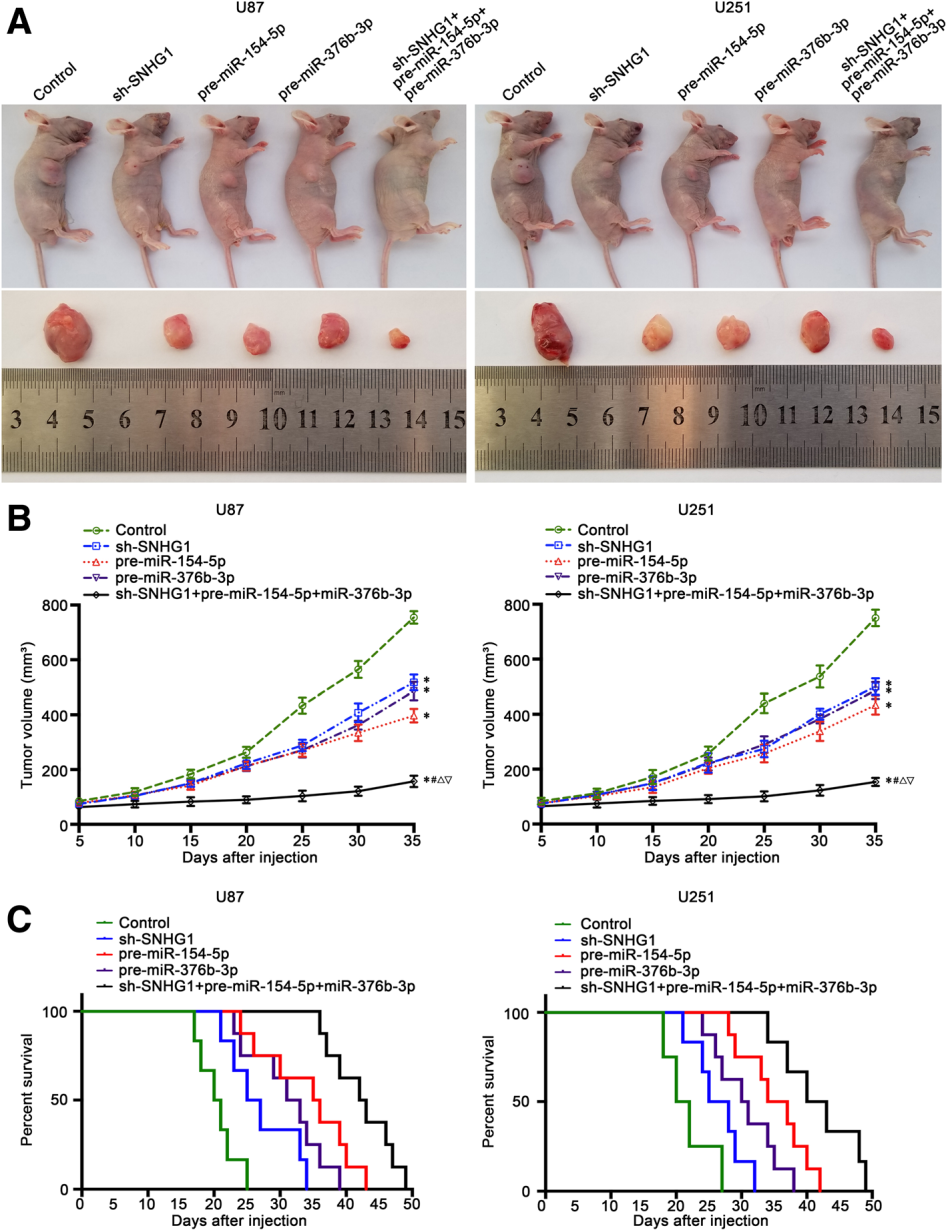
Tumor xenografts study of tumor growth and survival rates in nude mice. **a** The nude mice carrying tumors of respective groups were shown. Sample tumors from respective group were shown. Tumor growth curves of five groups in nude mice (*n* = 8, each group). **b** Tumor volume was calculated every five days after injection. Tumors were harvested on day 35 and weighed. **c** The survival curves of nude mice injected into the right striatum (*n* = 8, each group). *P < 0.05, ^*#*^*P* < 0.01, ^∆^*P* < 0.05, ^∇^*P* < 0.05

The effect of SHNG1, miR-154-5p and miR-376b-3p on glioma was further detected by xenograft tumor model in nude mice, dividing into five groups: control group, sh-SHNG1 group, pre-miR-154-5p group, pre-miR-376b-3p group and sh-SHNG1+ pre-miR-154-5p + pre-miR-376b-3p group. The results showed that compared with the control group, the volume of transplanted tumor in the sh-SHNG1 group, pre-miR-154-5p group and pre-miR-376b-3p group was significantly reduced, while the volume of sh-SHNG1 + pre-miR-154-5p + pre-miR-376b-3p group was smaller than that of the sh-SHNG1, pre-miR-154-5p, and pre-miR-376b-3p groups (Fig. 7a, b). The survival analysis was consistent with the results of subcutaneously transplanted tumors. Compared with the control group, the sh-SHNG1, pre-miR-154-5p, and pre-miR-376b-3p groups had longer survival time, whereas the survival time of the sh-SHNG1+ pre-miR-154-5p + pre-miR-376b-3p group was the longest, longer than that of the sh-SHNG1, pre-miR-154-5p, and pre-miR-376b-3p groups (Fig. 7c).

## Discussion

A large number of studies have confirmed that lncRNA plays an important biological role in the occurrence and development of many kinds of tumor diseases, as the molecular classification of adult diffuse gliomas being gradually recognized in recent years, lncRNA has been shown to determine a number of important cancer phenotypes by interacting with other cell macromolecules (including DNA, proteins and RNA) in related studies [2, 3, 37]. This analysis illustrated that SNHG1 acts as a tumor promoting factor in glioma tissues and cells. Similar to the results of this study, SNHG1 is highly expressed in colorectal cancer, cervical cancer, lung cancer, and other tissues and cells, playing a role in promoting cancer factors, silencing the expression of SNHG1, reducing the proliferation, migration, and invasion of corresponding tumor cells, and other malignant biological behaviors [7–9, 38, 39]. SNHG1 also can be used as a biological marker of poor prognosis [10, 40]. In glioma research, SNHG1 has been regarded as a cancer promoting factor that leads to the biological behavior of malignant glioma; decreasing the expression of SNHG1 can reduce the proliferation and invasion of glioma cells, whereas increasing cell apoptosis and SNHG1 expression in glioma tissues is correlated with poor prognosis. However, the potential mechanism of the biological effects of SNHG1 and other cellular molecules has not been further studied [11].

The research further demonstrated that miR-154-5p and miR-376b-3p play a role in tumor suppressor in glioma tissues and cells respectively. Similar to the results of this study, miR-154 is lowly expressed in prostate cancer, gastric cancer, non-small cell lung cancer, and other tissues and cells. The overexpression of miR-154 reduced the proliferation, migration, and invasion of tumor cells, and confirmed its antitumor effect [14–16, 41]. In the current study of glioma, miR-154-5p combined with PIWIL1 3 ‘UTR inhibits malignant progression of glioblastoma, the overexpression of miR-154-5p can inhibit proliferation and metastasis of malignant glioma cells and induce apoptosis; miR-154 can also be used as a potential biomarker for clinical prognosis in patients with glioma [13, 42]. The expression of miR-376b decreased significantly in glioma patients, and it can be used as an independent prognostic factor for glioma patients [19].

In this study, the expression of miR-154-5p and miR-376b-3p was negatively correlated with the expression of SNHG1 by the TCGA database, and the Starbase database predicted that there was a binding site between miR-154-5p and SNHG1, as well as between miR-376b-3p and SNHG1. The research showed that SNHG1 was combined with miR-154-5p and miR-376b-3p, and the silenced SNHG1 enhanced their expression. These results suggest that silencing the expression of SNHG1 can inhibit the proliferation, migration, and invasion of glioma cells by increasing the expression of miR-154-5p and miR-376b-3p, and promote the apoptosis of glioma cells. In addition, the RIP experiments confirmed that SNHG1 and miR-154-5p or miR-376b-3p existed in the RISC complex, and the study of SNHG1 as a miRNAs sponge binding to miRNAs and affecting the biological behavior of tumor cells has also been sighted in some reports. For example, silencing the expression of SNHG1, reducing the binding effect, increasing the expression of miR-199a-3p, and inhibiting the proliferation of prostate cancer cells [40]. In addition, lncRNA may participate in RNA precursor editing and splicing, in which lncRNA regulates the hydrolysis of adenosine from the double stranded RNA substrate to inosine, which is known as A to I editing [43]. A to I editing directed miRNAs to silence target genes, and existing studies have shown that the weakening of miR-376a* A to I editing in miR-376 family promotes the invasive ability of malignant glioma cells [20, 21]. MiR-376b-3p has a higher frequency of A to I editing in normal brain tissues and glioma tissues [44]. This study confirms that SNHG1 interacts with miR-376b-3p, which is edited by A to I, and plays a sponge regulating effect of the miRNAs molecule, suggesting that SNHG1 and miR-376b-3p can produce stable effects in glioma cells. However, whether SNHG1 is involved in editing miR-376b-3p A to I and redirecting miR-376b-3p to silence target genes is yet to confirm. This experiment only confirms that upon editing the miR-376b-3p A to I, the effect of targeting and binding FOXP2 3 ‘UTR region did not change, further demonstrating the conservative mechanism of SNHG1-miR-376b-3p-FOXP2 in the pathogenesis of glioma.

FOXP2 belongs to the fork box transcription factor family, which is expressed in many tissues, especially in brain development and maturation [22]. Although FOXP2 is a transcription factor closely related to neural development, a number of studies have confirmed that FOXP2 is involved in the development of many other tumor tissues, and whether FOXP2 is a tumor suppressor or cancer promoter remains controversial [23–26]. This research displayed that FOXP2 was highly expressed in glioma tissues and cells, and increased with the pathological grade of glioma. The immunohistochemistry results showed that the expression of FOXP2 in cytoplasm increased with the pathological grade, which was similar to the decreasing cytoplasmic expression of transcription factor ZEB1 in tumor tissues of patients with low grade esophageal cancer [45]. Silencing the expression of FOXP2 inhibits the proliferation, migration, and invasion of glioma cells and promotes apoptosis; the overexpression of FOXP2 has the opposite effect. The results suggest that FOXP2 acts as a tumor promoting gene in glioma tissues and cells. Further experiments showed that miR-154-5p and miR-376b-3p had targeted binding with 3 ‘UTR region of FOXP2, respectively. The experiment shows that the overexpression of miR-154-5p or miR-376b-3 inhibits proliferation, migration, and invasion of malignant glioma and promotes apoptosis; the overexpression of FOXP2 promotes proliferation, migration, and invasion of malignant glioma and inhibits apoptosis; only the overexpression of 3 ‘UTR wild type FOXP2 can reverse the inhibition of proliferation, migration, and invasion of glioma cells mediated by overexpressing miR-154-5p or miR-376b-3p, and also promote apoptosis of the glioma cells. These results suggest that the overexpression of miR-154-5p and miR-376b-3p may negatively regulate the biological behavior of malignant glioma by increasing the binding-to-target gene FOXP2 3’UTR. In combination with silencing SNHG1, the overexpression of miR-154-5p or miR-376b-3p inhibited the expression of FOXP2, whereas double silencing of SNHG1 and miR-154-5p or double silencing of SNHG1 and miR-376b-3p reversed these effects, suggesting that SNHG1 can be used as a molecular sponge of miR-154-5p or miR-376b-3p, weakening the negative regulation of RISC by miR-154-5p or miR-376b-3p and AGO2 protein complex on FOXP2, as a tumor promoting factor affecting the biological behavior of glioma.

KDM5B can produce histone 4 lysine (H3K4me3 and H3K4me2) demethylation of histone H3, thereby regulating the epigenetic changes of individuals [27, 28]. Many studies have confirmed that KDM5B is involved in the pathogenesis of multiple tissue tumors [29–31]. The expression of KDM5B can enhance the tumorigenicity and drug resistance of neuroblastoma [32]. This study proved that KDM5B was highly expressed in glioma tissues and cells, and increased with the pathological grade of glioma. Silencing the expression of KDM5B inhibits the proliferation, migration, and invasion of glioma cells and promotes apoptosis; the overexpression of KDM5B has the opposite effect. The results suggest that KDM5B acts as a tumor promoting gene in glioma tissues and cells. The results of KDM5B as a tumor promoting gene were consistent with the previous reports of Dai and Fan. KDM5B was highly expressed in the tissues and blood of glioma, and was positively correlated with the poor prognosis of patients [46, 47]. The results of this research further clarify the biological effects of KDM5B on glioma cells. Based on the prediction of bioinformatics software (JASPAR), this research explained that FOXP2 can enhance the transcriptional activity of KDM5B promoter region and bind to the KDM5B promoter region. The study also found that the overexpression of miR-154-5p and miR-376b-3p inhibited the expression of KDM5B, and attenuated the proliferation, migration, and invasion of glioma cells, promoting apoptosis; the overexpression of FOXP2, the increased abundance of KDM5B, enhance glioma cell proliferation, migration, and invasion ability, and reduce apoptosis; the overexpression of miR-154-5p and 3 ‘UTR wild-type FOXP2, as well as overexpression of miR-376b-3p and 3’ UTR wild-type FOXP2, respectively reversed the effect of overexpression of miR-154-5p and miR-376b-3p on the biological behavior of glioma cells. The results suggest that overexpression of miR-154-5p or miR-376b-3p can negatively regulate the expression of FOXP2, thereby affecting the transcription of target gene KDM5B by FOXP2, changing the expression of KDM5B, and inhibiting the malignant biological behavior of glioma cells.

The PI3K/Akt pathway is a classic signaling pathway involved in cancer progression, which can promote the proliferation, migration, and invasion of multiple tumor cells [48]. In vivo and in vitro studies have demonstrated that PI3K/Akt pathway inhibitors can inhibit the malignant biological behavior of glioma cells [49]. This study illustrated that the overexpression of KDM5B increases p-PI3K/PI3K, p-Akt/Akt, and also increases the activity of PI3K/Akt pathway, promotes the proliferation, migration, and invasion of glioma cells, and inhibits apoptosis; The expression of silent KDM5B inhibits p-PI3K/PI3K, p-Akt/Akt, namely, inhibits the activity of PI3K/Akt pathway, and also inhibits the proliferation, migration, and invasion of glioma cells, and promotes apoptosis. These results suggest that KDM5B can also promote the biological behavior of malignant glioma through the PI3K/Akt pathway.

The role of KDM5B in the demethylation of specific histone proteins can dynamically regulate the gene transcription process. It has been found that lncRNAs can participate in epigenetic modification by regulating histone modification enzymes [50, 51]. In KDM5B family, KDM4D can act as RNA binding protein in DNA repair, and KDM4D-RNA interaction is crucial for its localization in chromatin and the effective demethylation of histone substrate H3K9me3 [52]. Current studies have strong proofs to predict that KDM5B can bind to lncRNA MALAT1 to produce carcinogenic effects [53]. It is assumed that there is some interaction between KDM5B and lncRNA SNHG1. Based on the prediction of bioinformatics software (RPISeq, catRAPID), this analysis demonstrates that KDM5B can specifically bind to SNHG1 and maintain the stability of SNHG1. Silencing the expression of KDM5B in cell experiments significantly reduced the expression of SNHG1 in glioma cells. KDM5B exerts the effect of RNA binding protein, acts on SNHG1 to form a positive feedback loop, and regulates the biological behavior of glioma cells.

Finally, by studying the nude mice experiments, we have proved that sh-SHNG1, miR-154-5p, miR-376b-3p, and sh-SHNG1 + miR-154-5p + miR-376b-3p could inhibit glioma cell tumor and prolong the survival period respectively, compared with the single application of sh-SHNG1, miR-154-5p, miR-376b-3p, the combination of the three factors had the smallest volume and longest survival time. The results suggest that the combined use of sh-SHNG1, miR-154-5p, and miR-376b-3p has potential clinical value.

## Conclusions

In summary, this study demonstrates that SNHG1 increases the expression of FOXP2 and KDM5B by regulating the expression of miR-154-5p and miR-376b-3p, and that KDM5B itself and its downstream PI3K/Akt pathway affect the biological behavior of glioma cells. SNHG1-miR-154-5p/miR-376b-3p-FOXP2-KDM5B feedback loop plays an important role in regulating the biological behavior of glioma cells. The results provide a basis for the mechanism of glioma development and targeted therapy of human glioma.

## Additional files

Additional file 1: **Figure S1.** SNHG1 also bound to pre-miR-376b-3p with A to I edited. Schematic representation of the putative binding site between SNHG1(SNHG1-Wt) (A) or 3′-UTR region of FOXP2 (FOXP2–3′-UTR-Wt) (B) and miR-376b-3p with A to I edited, and the designed mutant sequence (SNHG1-Mut, FOXP2–3′UTR-Mut) indicated for the dual-luciferase reporter assay. Renilla/firefly luciferase ratios were calculated and further normalized. **P < 0.01 vs. pre-NC group. (JPG 1617 kb)

Additional file 2: **Figure S2.** (A) SNHG1 knockdown and miR-154-5p or miR-376b-3p overexpression suppressed the glioma cells proliferation. (B) SNHG1 knockdown and miR-154-5p or miR-376b-3p overexpression increased the glioma cells apoptosis. (C) SNHG1 knockdown and miR-154-5p or miR-376b-3p overexpression inhibited migration and invasion of U87 and U251 cells. Scale bars represented 20 μm. For A, B and C, data were presented as the mean ± SD (*n* = 5, each group). **P < 0.01 vs. sh-NC + pre-NC group, ^*##*^*P* < 0.01 vs. sh-SNHG1 + pre-miR-154-5p group, ^∆∆^*P* < 0.01 vs. sh-SNHG1 + pre-miR-376b-3p group. (D) FOXP2–3′-UTR-Wt reversed overexpression of miR-154-5p and miR-376b-3p induced inhibition of glioma cells proliferation. (E) FOXP2–3′-UTR-Wt reversed overexpression of miR-154-5p and miR-376b-3p induced augmentation of glioma cells apoptosis. (F) FOXP2–3′-UTR-Wt reversed overexpression of miR-154-5p and miR-376b-3p induced reduction of migration and invasion of U87 and U251 cells. Scale bars represented 20 μm. For D, E and F, data were presented as the mean ± SD (*n* = 5, each group). **P < 0.01 vs. pre-NC + FOXP2-NC group, ^*##*^*P* < 0.01 vs. pre-miR-154-5p + FOXP2-NC group, ^∆∆^*P* < 0.01 vs. pre-miR-376b-3p + FOXP2-NC group. (TIF 14809 kb)

## Abbreviations

CHIP: Chromatin immunoprecipitation
FOXP2: Fork-head box protein P2
GBM: Glioblastoma multiforme
HGG: High-grade glioma group
IHC: Immunohistochemistry staining
KDM5B: Histone lysine specific demethylase (5B lysine (K) -specific demethylase 5B)
LGG: Low-grade glioma group
lncRNAs: Long non-coding RNAs
NBTs: Normal brain tissues
qRT-PCR: Quantitative real-time polymerase chain reaction
RIP: RNA immunoprecipitation
SNHG1: Small nucleolar RNA host gene 1
TCGA: The Cancer Genome Atlas
