# Supplementary figures and images for: SNHG1 promotes malignant biological behaviors of glioma cells via microRNA-154-5p/miR-376b-3p- FOXP2- KDM5B participating positive feedback loop
Source: J Exp Clin Cancer Res. 2019 Feb 6;38:59. doi: 10.1186/s13046-019-1063-9 (PMC6364475; doi:10.1186/s13046-019-1063-9)

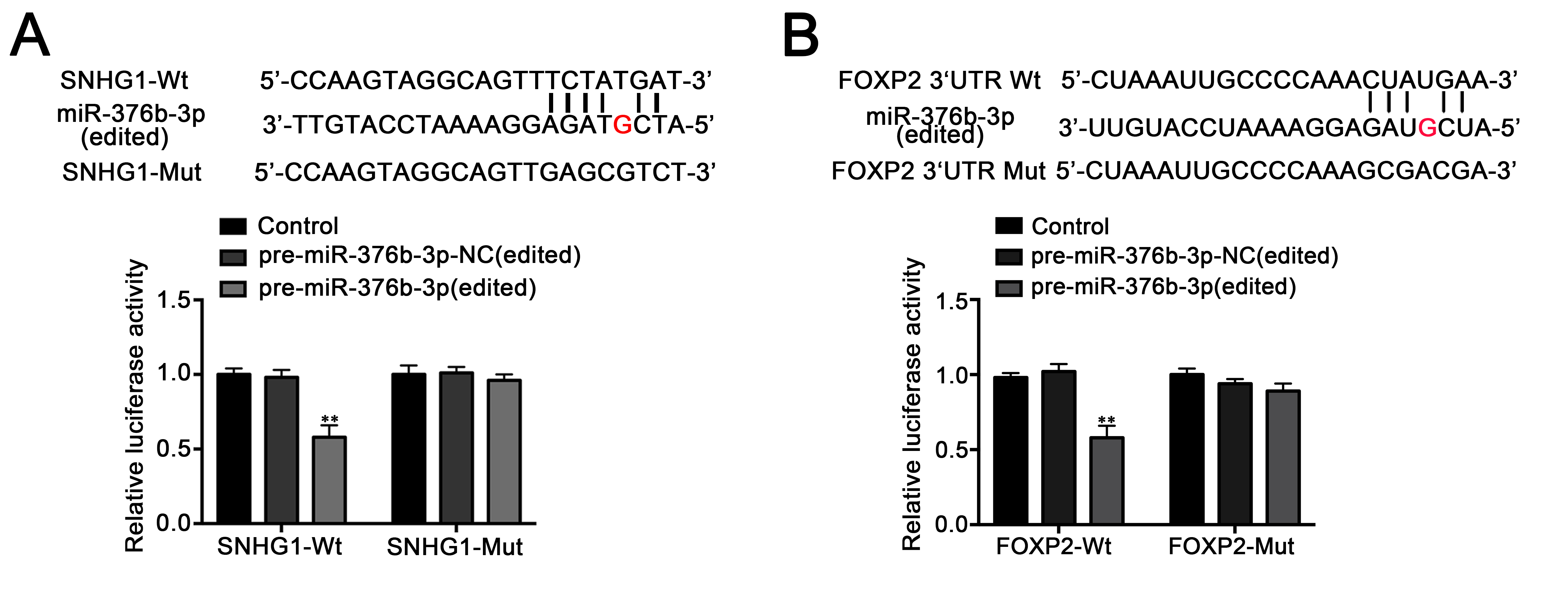

Supplement: Supplementary file 1 — Figure S1. SNHG1 also bound to pre-miR-376b-3p with A to I edited. Schematic representation of the putative binding site between SNHG1(SNHG1-Wt) (A) or 3′-UTR region of FOXP2 (FOXP2–3′-UTR-Wt) (B) and miR-376b-3p with A to I edited, and the designed mutant sequence (SNHG1-Mut, FOXP2–3′UTR-Mut) indicated for the dual-luciferase reporter assay. Renilla/firefly luciferase ratios were calculated and further normalized. **P < 0.01 vs. pre-NC group. (JPG 1617 kb) [file 13046_2019_1063_MOESM1_ESM.jpg]

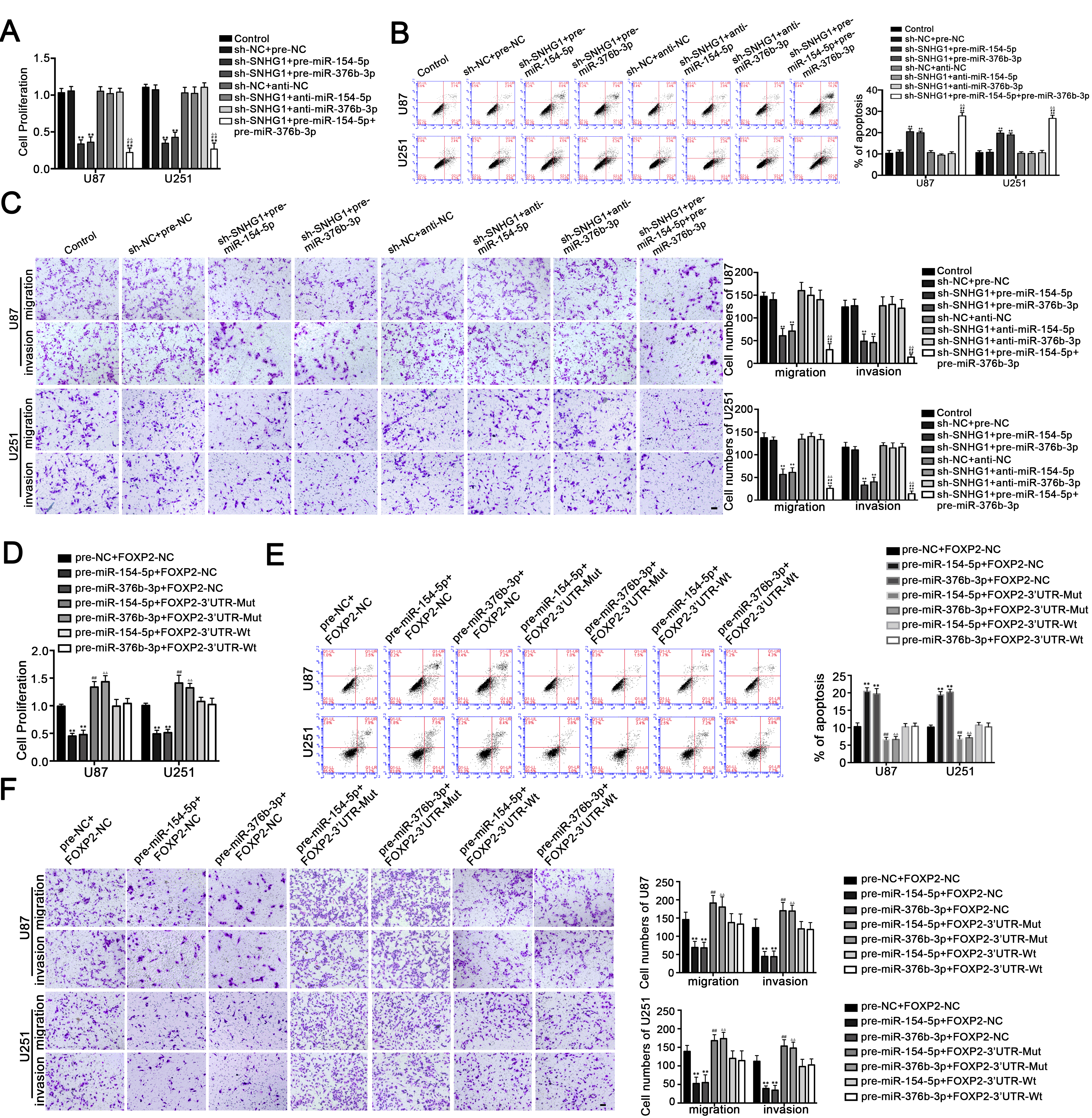

Supplement: Supplementary file 2 — Figure S2. (A) SNHG1 knockdown and miR-154-5p or miR-376b-3p overexpression suppressed the glioma cells proliferation. (B) SNHG1 knockdown and miR-154-5p or miR-376b-3p overexpression increased the glioma cells apoptosis. (C) SNHG1 knockdown and miR-154-5p or miR-376b-3p overexpression inhibited migration and invasion of U87 and U251 cells. Scale bars represented 20 μm. For A, B and C, data were presented as the mean ± SD (n = 5, each group). **P < 0.01 vs. sh-NC + pre-NC group, ##P < 0.01 vs. sh-SNHG1 + pre-miR-154-5p group, ∆∆P < 0.01 vs. sh-SNHG1 + pre-miR-376b-3p group. (D) FOXP2–3′-UTR-Wt reversed overexpression of miR-154-5p and miR-376b-3p induced inhibition of glioma cells proliferation. (E) FOXP2–3′-UTR-Wt reversed overexpression of miR-154-5p and miR-376b-3p induced augmentation of glioma cells apoptosis. (F) FOXP2–3′-UTR-Wt reversed overexpression of miR-154-5p and miR-376b-3p induced reduction of migration and invasion of U87 and U251 cells. Scale bars represented 20 μm. For D, E and F, data were presented as the mean ± SD (n = 5, each group). **P < 0.01 vs. pre-NC + FOXP2-NC group, ##P < 0.01 vs. pre-miR-154-5p + FOXP2-NC group, ∆∆P < 0.01 vs. pre-miR-376b-3p + FOXP2-NC group. (TIF 14809 kb) [file 13046_2019_1063_MOESM2_ESM.tif]
